# Supplementary figures and images for: Loss of Cdc42 causes abnormal optic cup morphogenesis and microphthalmia in mouse
Source: Front Cell Neurosci. 2024 Nov 20;18:1474010. doi: 10.3389/fncel.2024.1474010 (PMC11622195; doi:10.3389/fncel.2024.1474010)

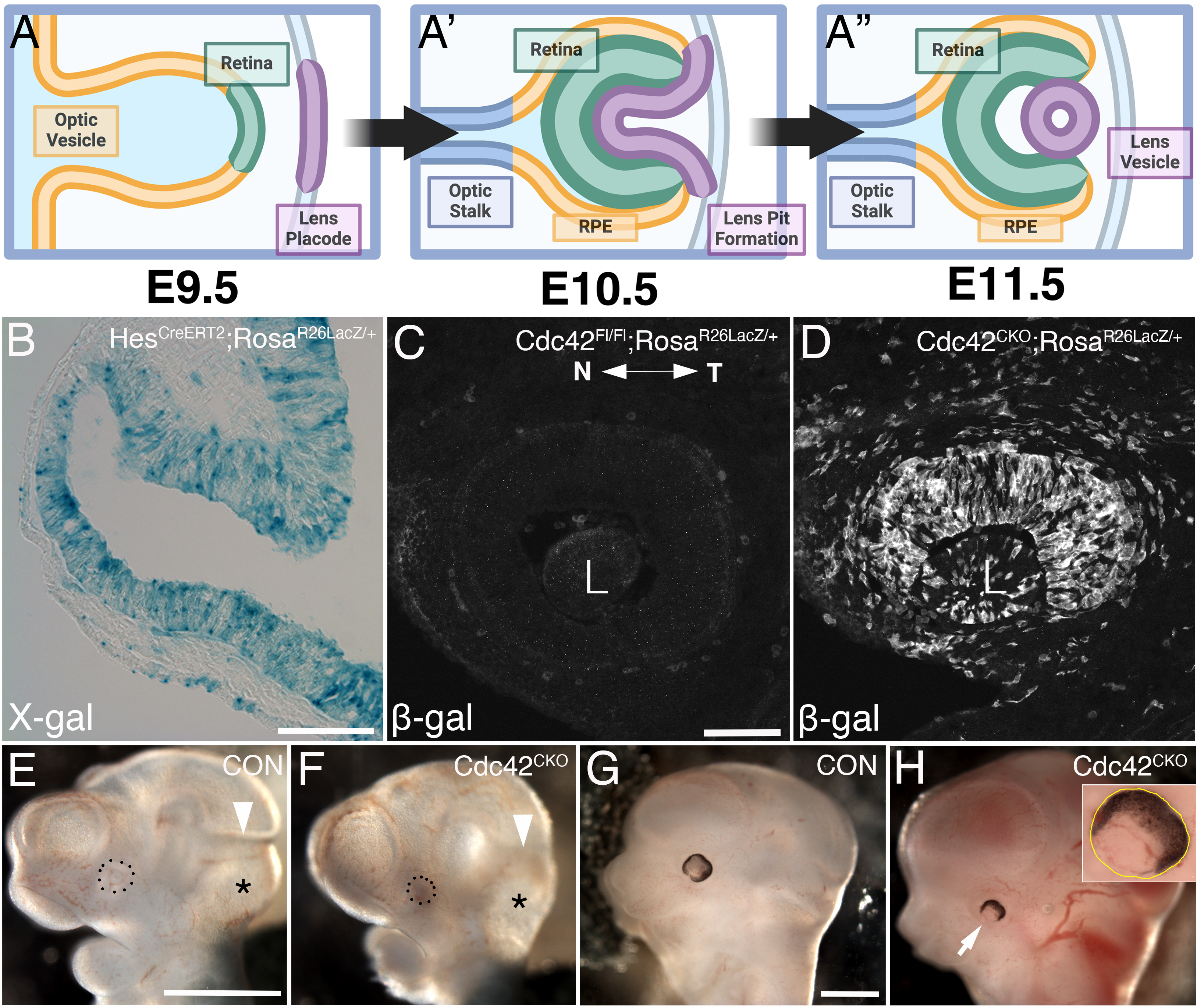

Supplement: SUPPLEMENTARY FIGURE S1 — General features of Cdc42 disruption induced between E7.5-E8.5. (A) Schematic view of formation of the optic vesicle, patterning of the retina distally and establishment of the lens placode (E9.5 in mouse). (A’) The optic vesicle and lens ectoderm invaginate, resulting in formation of optic cup and lens pit, with concomitant patterning of the optic stalk (E10.5 in mouse). (A”) Separation of the lens vesicle (E11.5 in mouse). (Created in Biorender.com) (B) X-gal-labeled E9.25 embryo (HesCreERT2;RosaR26FL/+), tamoxifen-induced at E8.0. The reporter is robustly activated in the optic vesicle. (B-H) Nasal and temporal orientation are left and right, respectively. (C,D) Beta-galactosidase labeling of control without Cre (C) and Cdc42CKO;RosaR26FL/+ reveal robust recombination in ocular tissues and mosaic expression in lens and extraocular mesenchyme in Cdc42CKO (D). (E,F) Heads of embryos harvested at E11.0, representative for tamoxifen administration between E7.5-E8.0. Eyes are marked by dots in control (E, Cdc42FL/FL, 33 somites) and in Cdc42CKO (F, 31 somites). In Cdc42CKO at E11.0, the hindbrain vesicle was smaller, and the midbrain-hindbrain boundary was not as distinct, compared to controls (E,F asterisks and arrowheads, respectively). (G,H) Embryonic heads at E11.5, representative for tamoxifen administration at E8.5. Cdc42CKO exhibit a wide coloboma (H, arrow). Inset in (H) shows outline of eye circumference used for determining ROI to calculate eye size in Cdc42CKO. L = lens. Scale bars B, C: 100 µm, E, G: 1 mm. [file Image_1.jpg]

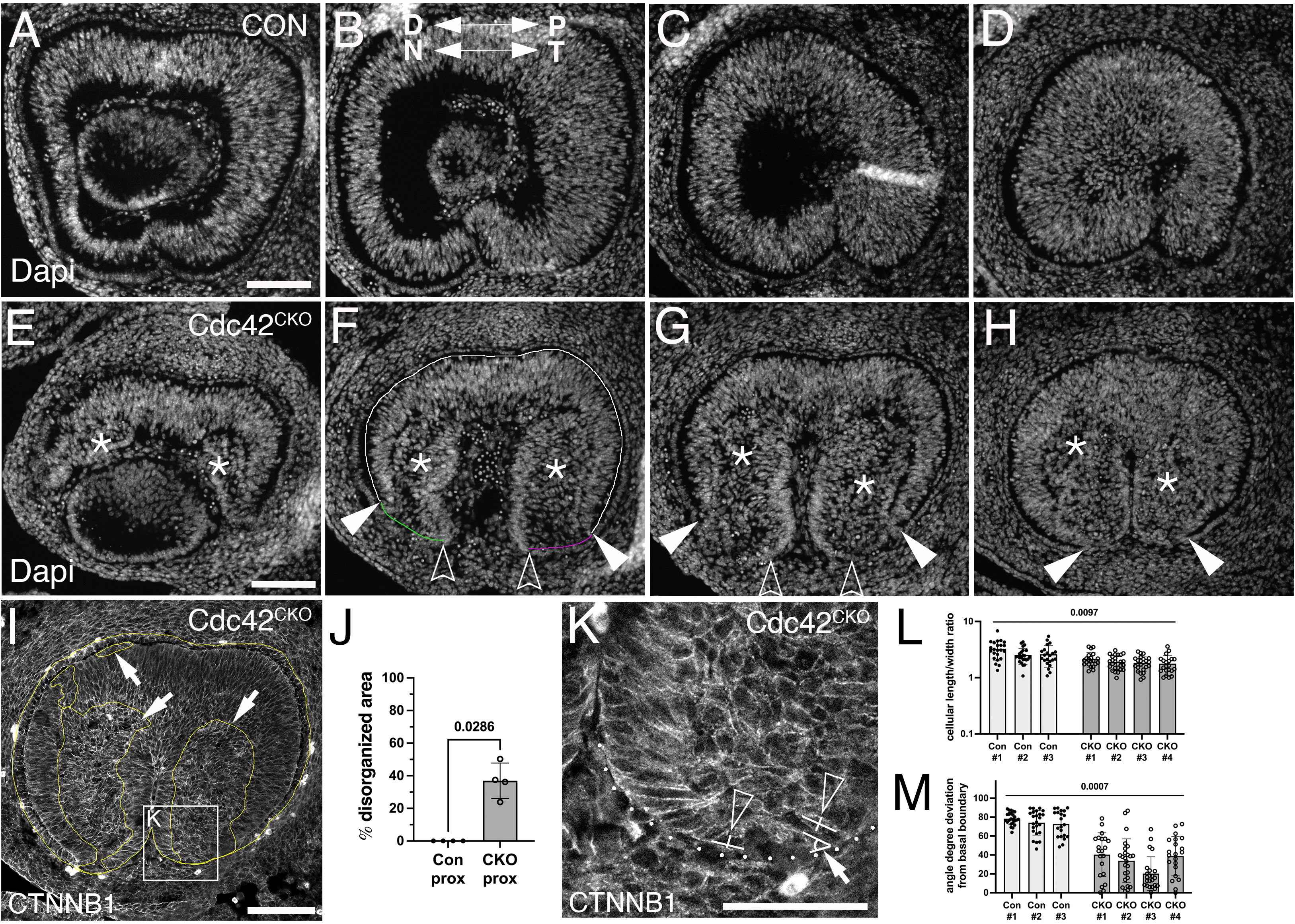

Supplement: SUPPLEMENTARY FIGURE S2 — (A-H) Dapi labeling of mostly sequential sections from distal to proximal levels of control (A-D) and Cdc42CKO optic cups of E12.5 embryos (E-H). (F-H) Arrowheads mark shortening of the subretinal space in the ventral optic cup of Cdc42CKO. Open arrowheads point to the outer, basal side of the optic cup. (F) Lines mark extent of apical boundaries/subretinal space (white), extent of shortening of temporal subretinal space (magenta) and nasal subretinal space (green) in the ventral Cdc42CKO optic cup. Example for disruption of retinal progenitor organization in the inner portion in Cdc42CKO optic cups (E-H; asterisks). (I) Examples of b-catenin-labeled disorganized regions in the proximal optic cup (arrows and yellow lines; includes eye circumference outline along the basal boundary of the optic cup). (J) Quantification of disorganized regions in controls (n=4 embryos, set as 0%) and Cdc42CKO (n=4 embryos), calculated as percent of the total eye area. Mann-Whitney test (p=0.0286). (K) Higher magnification of boxed area shown in (J). Examples for measurements of cell shape and alignment of cells in the defective ventral optic cup that lacks subretinal space formation and RPE patterning defects. (L) Quantification of cell shape in control (n=3) and Cdc42CKO embryos (n=4). Length and width of 21-25 cells per embryo were measured. Data are shown with SEM (nested T-test, p=0.0097). (M) Quantification of cell alignment in control (n=3) and Cdc42CKO embryos (n=4). The angle between the length of each cell and the basal boundary of the optic cup (set as 0 degrees) was determined for 21-25 cells per embryo. Data are shown with SEM (nested T-test, p=0.0007). Scale bars A, E, I: 100 µm, K: 50 µm. [file Image_2.jpg]

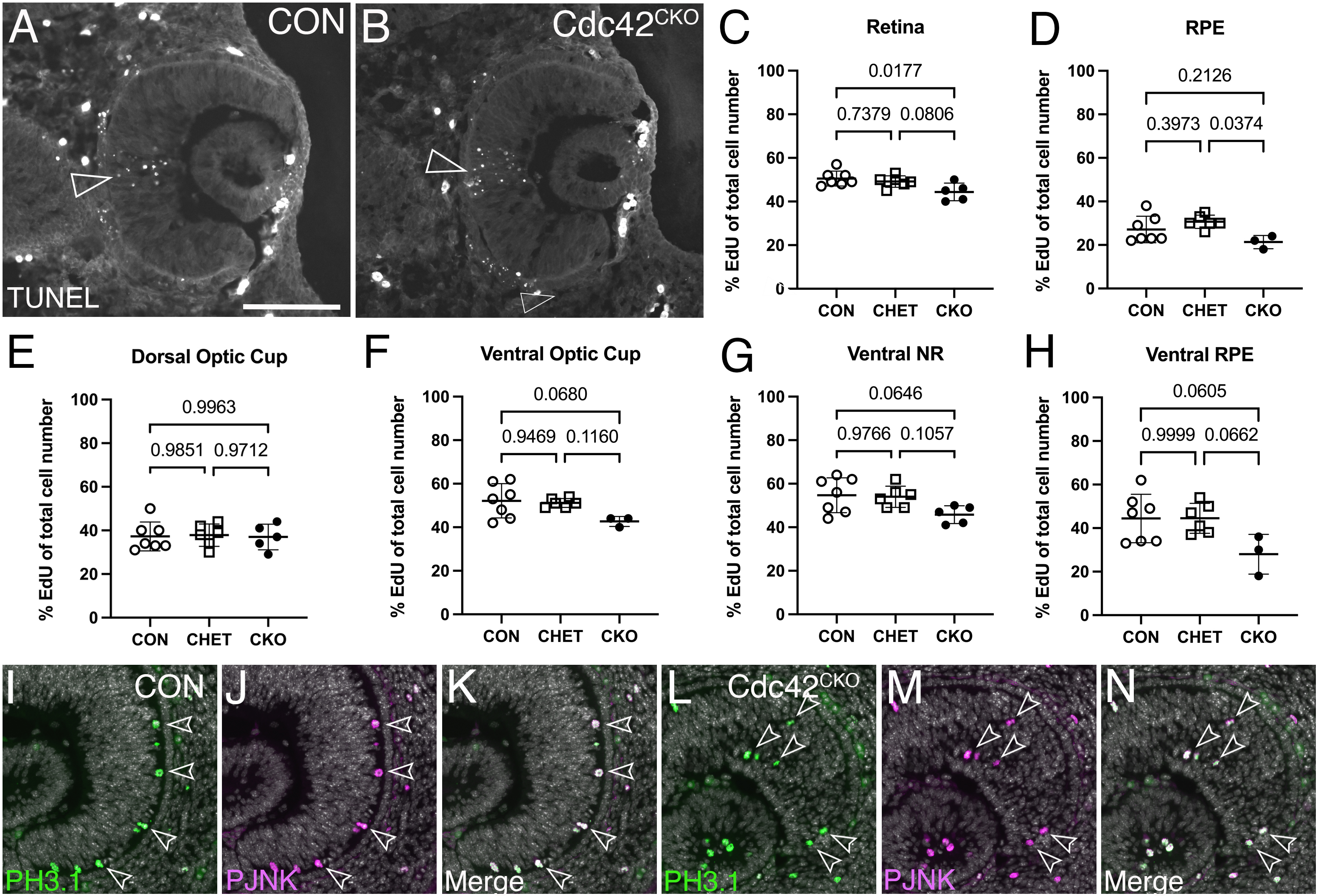

Supplement: SUPPLEMENTARY FIGURE S3 — EdU incorporation is reduced in the ventral optic cup, and PH3.1-positive retinal progenitors co-express pJNK. (A,B) Coronal view of TUNEL labeling of control (A, Cdc42FL/FL) and Cdc42CKO eyes (B). (C-H) Quantitative analyses of cells incorporating EdU in Cdc42FL/FL controls, Cdc42HET and Cdc42CKO retina (C), RPE (D), dorsal optic cup (E), ventral optic cup (F), ventral retina (G) and ventral RPE (H). A general trend in decrease of EdU-labeled cells is detectable in all domains, except in the dorsal optic cup. For embryo number analyzed, see Legend for Figure 4A. Each data point represents n=1 embryo (one-way ANOVA with Tukey’s posthoc analysis). (I-N) Colocalization of PH3.1 (green) and PJNK (magenta) in retinal progenitors at the apical border in Cdc42FL/FL controls (I-K; arrowheads) and in mis-localized progenitors in Cdc42CKO (L-N; arrowheads). Scale bar: 100 µm. [file Image_3.JPEG]
